# Detection of SRY‐positive46,XX male syndrome by the analysis of cell‐free fetal DNA via non‐invasive prenatal testing

**DOI:** 10.1002/ccr3.2389

**Published:** 2019-09-07

**Authors:** Luigia De Falco, Giovanni Savarese, Teresa Suero, Sonia Amabile, Raffaella Ruggiero, Pasquale Savarese, Antonio Fico

**Affiliations:** ^1^ AMES, Centro Polidiagnostico Strumentale Srl Naples Italy; ^2^ Department of Molecular Medicine and Medical Biotechnology University of Naples "Federico II" Naples Italy

**Keywords:** aneuploidies, circulating cell‐free fetal DNA, non‐invasive prenatal testing, sex discordance

## Abstract

We report a new case of 46,XX male syndrome that was detected following an anomalous result by non‐invasive prenatal testing (NIPT) and a discrepancy between the fetal karyotype and the ultrasonographic investigation. With the increasing use of NIPT, more gender discordances can be identified prenatally and be amenable to early therapy.

## 1 INTRODUCTION

Since the identification of circulating cell‐free fetal DNA (cffDNA) in maternal blood, non‐invasive prenatal testing (NIPT) by massive parallel sequencing (MPS) has permitted the early diagnosis of common fetal aneuploidies with high specificity and sensitivity.^1^, ^2^ In addition, NIPT using MPS can identify fetal sex aneuploidies (SCAs), although with a lower accuracy due to biological limits and little clinical experience.^3^, ^4^ Although maternal and fetal biological features could affect fetal sex aneuploidy detection,^5^ disorders of sexual differentiation (DSD) may also produce a discordance between the NIPT sex genotype and ultrasonographic detection or fetal karyotype. DSD is found in approximately 1/2500 pregnancies.^6^ 46,XX male syndrome, which was first described in 1964 by de la Chapelle, has a prevalence of 1:20 000 in newborn males,^7^ with different clinical phenotypes, such as normal or ambiguous male external genitalia, infertility, or hypogonadism, presenting at birth or later during puberty.^8^ Approximately 90% of cases show the presence of the sex‐determining region Y (SRY) and are usually diagnosed after puberty when they develop hypogonadism, gynecomastia, and/or infertility.^9^ SRY‐negative subjects (10%) usually have ambiguous external genitalia to a variable degree, although a complete male phenotype can also be observed.^10^

In this paper, we described a novel case of 46,XX male syndrome arising from a sex discrepancy between NIPT results, with a partial Y chromosome detection, and the ultrasound examination of the fetus. Informed consent was obtained before performing these studies.

### 1.1 Clinical report

A 40‐year‐old pregnant Caucasian woman at 11 + 2 weeks of gestation arrived to our center without particular indications except for maternal age. Considering the risk of invasive testing, the patient chose to undergo NIPT analysis. We performed whole genome sequencing (WGS) for the detection of aneuploidies of chromosomes 13, 18, 21, X, and Y using cffDNA from the maternal blood.^11^ No aneuploidies were observed for chromosomes 21, 18, and 13 and two X chromosomes and a partial Y chromosome, which is not completely consistent with a Klinefelter karyotype, were detected. The test was repeated at 14 + 3 weeks of gestational age, confirming our previous findings. A routine fetal morphology assessment at 18 weeks showed no structural anomalies and external genitalia consistent with a male. Because of the discordant result between NIPT and the ultrasound screening, we suggested performing further investigations including cytogenetic analyses on the amniotic fluid. GTG‐banding and quantitative fluorescent polymerase chain reaction (QF‐PCR) analysis of chromosomes 13, 18, 21, X, and Y as well as cytogenetic molecular studies were performed.

## 2 MATERIALS AND METHODS

### 2.1 NIPT analysis

A blood sample was collected from the pregnant woman at AMES Laboratory. cffDNA was extracted from 900 μL of maternal plasma using the QIAamp DNA Blood Mini Kit (Qiagen) following the manufacturer's protocol. The pipeline included automated library preparation (VeriSeq NIPT Solution, Microlab STAR, Illumina) and WGS sequencing on a Next550 (Illumina). The VeriSeq NIPT Assay Software (www.illumina.com/NIPTsoftware) was used for data analysis of the aneuploidy status and fetal fraction from cffDNA. Briefly, generated WGS data were streamed to the VeriSeq NIPT Analysis Server where the software filtered and aligned the WGS reads to a reference genome. The software used a sophisticated counting‐based algorithm to generate log likelihood ratio (LLR) scores for each sample for each of the five tested chromosomes, including 13, 18, 21, X, and Y. We performed clinical validation studies to establish LLR thresholds. The final results for each sample arise from the evaluation of LLR scores that rely on our clinical validation (personal data).

### 2.2 Karyotype analysis

Amniotic fluid was drawn, and genomic DNA was extracted from the amniocyte using the QIAamp DNA blood Mini Kit (Qiagen). Aneuploidies for chromosomes 13, 18, and 21 and the sex chromosomes were first screened by quantitative fluorescence polymerase chain reaction (QF‐PCR;Devyser Compactv3, Devyser) as previously described.^12^ The amplified DNA samples were separated by electrophoresis using an ABI 3130xl Genetic Analyzer, and the analysis of each allele for specific markers was performed using the GeneMapper Software ver. 4.0 (Applied Biosystems). GTG‐banding analysis of amniotic fluid was performed in established cell culture following standard laboratory protocols. Metaphases were analyzed with the CytoVision software (CytoVision, AB Imaging).

### 2.3 Molecular cytogenetic analyses

Fluorescent in situ hybridization (FISH) was performed on both interphase nuclei and metaphase chromosomes from amniotic fluid. A probe mix containing the SRY gene (Yp11.31, locus‐specific probe, labeled in red), X centromere (DXZ1, labeled in aqua), and chromosome Y (DYZ1, the heterochromatic block at Yq12, labeled in green) (Cytocell, Oxford Gene Technology) was used to detect SRY translocation. Probe mix containing probes for the chromosome 8 centromere (8p11.1‐q11.1, labeled in orange; Cytocell, Oxford Gene Technology), and chromosome 17 centromere (17p11.1‐q11.1, labeled in green; Cytocell, Oxford Gene Technology) was used to define the small supranumerary marker chromosome (sSMC) origin. FISH slides were examined using a LEICA DM5500 B fluorescence microscope and analyzed with the CytoVision software (CytoVision, AB Imaging).

Single Nucleotide Polymorphism (SNP) array analysis was performed using a HumanCytoSNP‐12v12.1 kit following the manufacturer's protocol (http://www.support.illumina.com/array/protocols.ilmn). Stained BeadChips were scanned with a HiScanSystem (Illumina). Data were generated with GenomeStudio (Illumina) and analyzed with the Bluefuse Multi Software (Illumina). All CNVs >100 Kb were interrogated. All results were reported according to the GRCh37 (hg19) assembly.

To confirm the presence of the Y chromosome, we used a commercial kit that allows for the analysis of chromosome Y microdeletions by real‐time PCR (Sacace Biotechnology Srl). The assay detected two STS loci in each AZF region (AZFa, AZFb, and AZFc) and a unique region in both male and female DNA (ZFX/ZFY) with two multiplex mixes.

## 3 RESULTS

**Figure 1:**
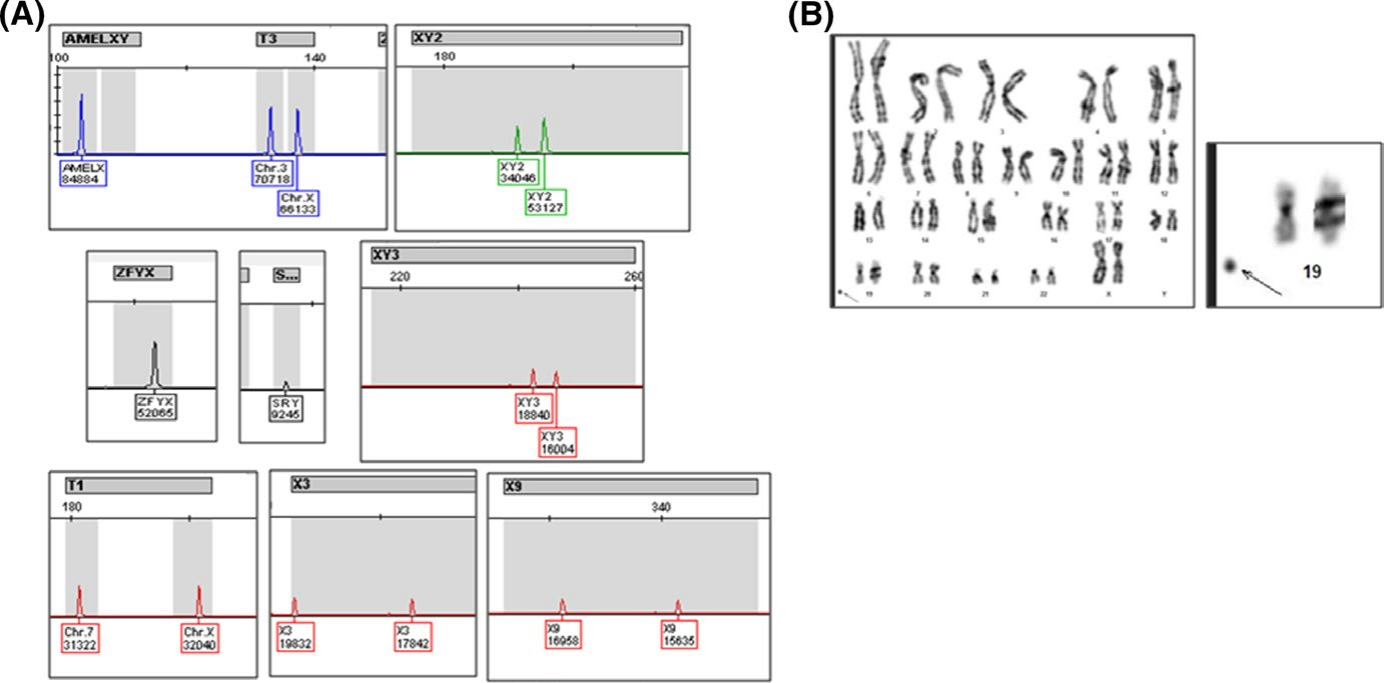
Conventional and molecular cytogenetics characterization of fetal amniotic fluid. A, quantitative fluorescent polymerase chain reaction (QF‐PCR) detected 46,XX with the presence of SRY. Two markers (DXS981,X9 and XHPRT,X3) have a pattern compatible with the presence of two X chromosomes: one (AMELX/AMELY) indicates the presence of an X chromosome(s) and the absence of a Y chromosome; and one (SRY) was compatible with the presence of the Y chromosome. The presence of an informative X chromosomal STR marker confirms the two X chromosome dosage. T1 and T3 markers (1:1 ratio between autosomal and X chromosomes) confirm the two X chromosome dosage. B, GTG‐banding analysis of amniotic fluid showed a female karyotype with a small supernumerary marker chromosome (sSMC, 47,XX+ mar)

**Figure 2:**
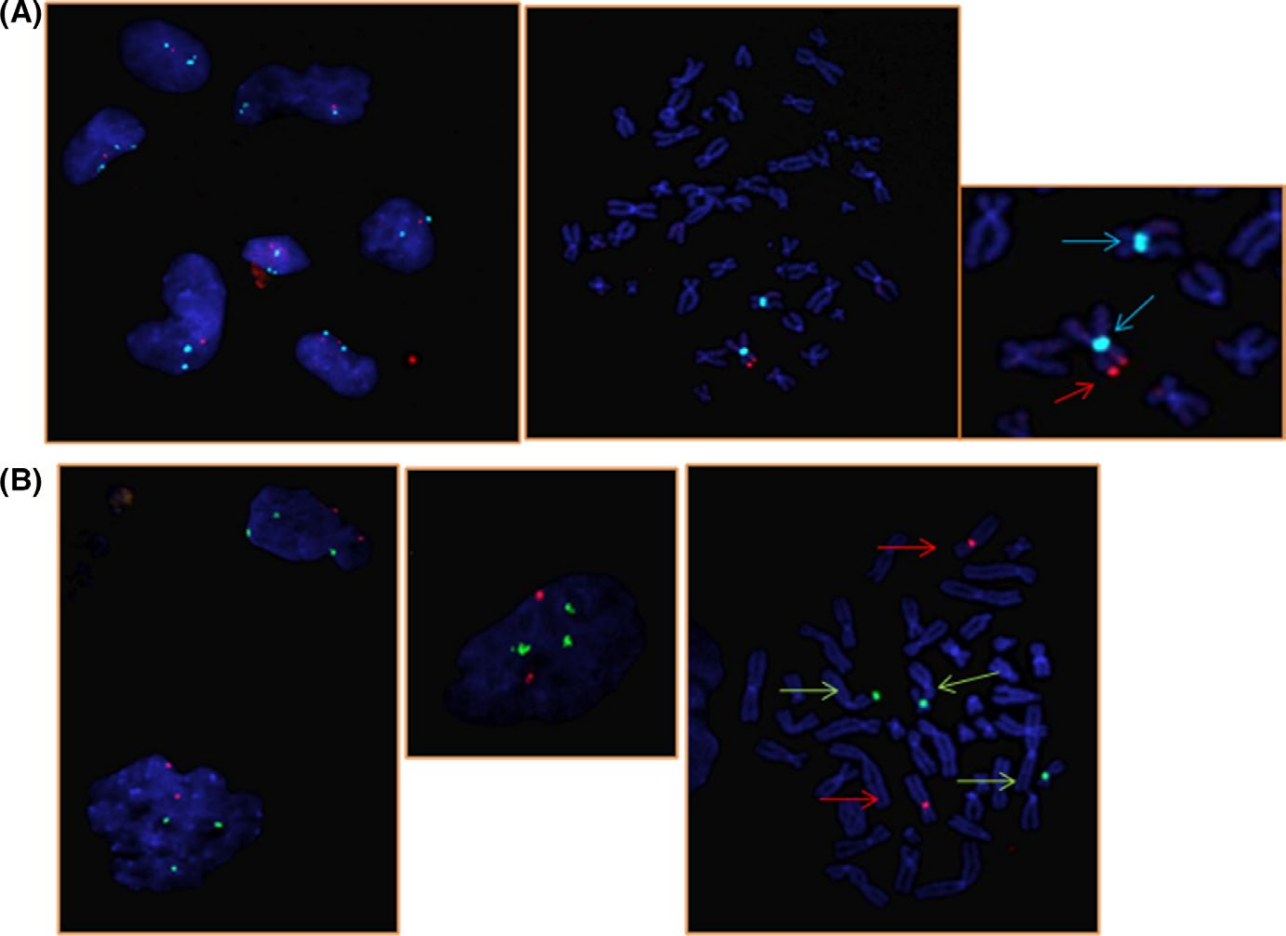
Fluorescent in situ hybridization (FISH) for both interphase nuclei and metaphase chromosomes from the amniotic fluid. A, A positive SRY signal indicated by the red arrow is found at the terminal derivative X p‐arm. The centromeres of both X chromosomes are shown by the light blue arrows. B, FISH analysis for the small supernumerary marker chromosome (sSMC) showed two signals for the chromosome 8 centromere (labeled in red), three signals for the chromosome 17 centromere (labeled in green) and defined the origin of the sSMC from chromosome 17

In this study, NIPT analysis was performed and no aneuploidies were detected in chromosomes 21, 18, and 13. We unexpectedly found the presence of two X chromosomes and a partial Y chromosome. During a follow‐up ultrasound examination of the fetus, the external genitalia were consistent with a male. The cffDNA test was repeated to exclude the false‐positive NIPT screening, but the presence of a Y chromosome was reported. The pregnant woman allowed us to further investigate these gender discordant results between the NIPT and ultrasound screening. QF‐PCR showed that the fetus was disomic for chromosomes 13, 18, 21, and X; moreover, a marker for *SRY* was reported (Figure 1A), confirming the genotype described by NIPT. In particular, all informative autosomal STR markers showed a normal 1:1 marker ratio (Figure 1A). The presence of an informative X chromosomal STR marker confirms the dosage of two X chromosomes. Fetal sex is assessed by the presence of the X chromosome‐specific AMXY product and the absence of a Y chromosome‐specific AMXY product. T1 and T3 markers in the same proportion generate a 1:1 peak ratio associated with a normal female. The SRY product was compatible with the presence of the Y chromosome. Additionally, Y microdeletion analysis of the fetal DNA confirmed the presence of both an X chromosome and an *SRY* marker as well as AZFa, AZFb, and AZFc regions (data not shown). Chromosomal analysis using GTG‐banding revealed a 47,XX karyotype (450 bands of resolution) and the presence of a sSMC based on a positive C‐banding result (Figure 1B). This latter feature was also present in the maternal karyotype from peripheral blood but was not found in paternal karyotype (data not shown). Fluorescence in situ hybridization (FISH) studies using a set of centromeric probes (Cytocell, Oxford Gene Technology, UK) enabled the identification of the sSMC 17 origin (Figure 2B).

Moreover, to confirm the observation of the *SRY* gene, FISH analysis was performed using SRY probes, including those for the *SRY* gene, the Y chromosome heterochromatin region, and the X chromosome centromere (SRY, DYZ1, and DXZ1, respectively) (Cytocell, Oxford Gene Technology; Figure 2A). We found a centromeric signal on both X chromosomes with an additional red signal on the p‐arm of only one X chromosome corresponding to the *SRY* gene.

**Figure 3:**
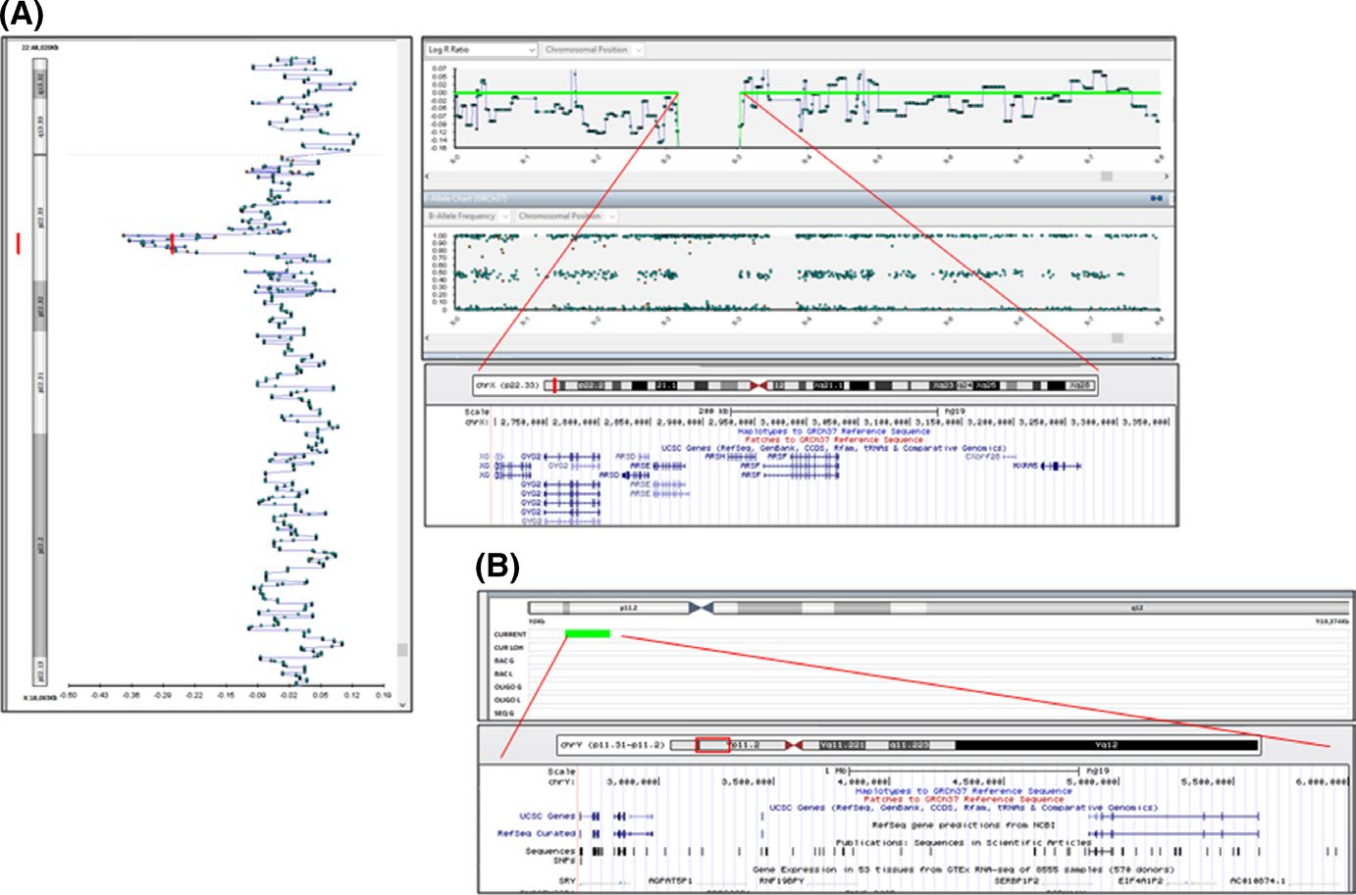
SNP array images of the (A) X and (B) Y chromosomes. The log ratio of 0 from Xp22.33 to Xp22.32 indicates a copy number loss of 670 Kb. The log ratio of 0 from Yp11.31 to Yp11.2 indicates a copy number gain of 3 Mb. The results were reported as follows: arr[GRCh37] (X)x2, Xp22.33 (2 697 868‐3 356 539)x1, Yp.11.31p11.2(2 654 900‐6 000 464)x1, and Yp.11.2(7 508 686‐9 111 897)x1. Stained BeadChips were scanned using a HiScan System (Illumina). Data were generated with GenomeStudio (Illumina) and analyzed with the Bluefuse Multi Software (Illumina). All CNVs > 100 Kb were interrogated

SNP array analysis (HumanHap550, Illumina Infinium) performed on amniotic fluid found a 680 Kb deletion at Xp.22.33 that included the *XG* [OMIM*300879] and *ARSE* [OMIM*300180] genes as well as the presence of a 3.5 Mb band at Yp.11.31p11.2 that included *SRY* [OMIM*480000] (Figure 3A, B).

## 4 DISCUSSION

46,XX male syndrome is a rare condition (1:20 000 in newborn males), and the presence of the *SRY* gene, which is due to a translocation of part of the Y chromosome to the X chromosome, is found in most patients (90%).^7^ Mutations in genes involved in testis differentiation such as *SOX9*, *SOX3,* and *RSPO1* and Y chromosome mosaicism are usually responsible for *SRY*‐negative cases.^13^ External genitalia and masculinization are usually normal in 46,XX *SRY*‐positive individuals, and the diagnosis is usually made later (adolescence or adulthood) due to infertility and/or the presence of a small testis during clinical investigation.^9^

In our case, we suspected a sex development disorder during prenatal age based on sex discordance between NIPT and ultrasound screening. Indeed, an anomalous X and Y pattern was already evident based on the NIPT results, since the sex chromosomes were not completely compatible with the XX or XXY karyotype. The analysis of “raw data” rather than the final report as “Aneuploidy detected” or “No Aneuploidy detected” allowed us to realize that there was a change in the sex chromosome pattern before ultrasound screening results confirmed a possible sex discordance between the NIPT gender test and the phenotypically male features. In particular, the observed Y chromosome in the “raw data” was lower than expected in a normal male and was considered inconclusive. The presence of the *SRY* gene alone would probably not have been sufficient for the VeriSeq NIPT Assay Software to determine there was at least a partial Y chromosome present.

Conventional cytogenetic analysis on amniotic fluid showed a 47,XX+ mar karyotype, while molecular cytogenetic techniques showed that the *SRY* gene translocated to the short arm of the X chromosome, which is compatible with 46,XX male syndrome. Supernumerary marker chromosomes, which are inherited from the mother, are derived from the centromeric region of chromosome 17. Any possible phenotype‐genotype correlation regarding the supernumerary marker chromosome was excluded in the mother. The clinical phenotypes associated with marker chromosomes are highly variable, ranging from normal to severely abnormal,^14^ and this renders marker chromosomes a particularly difficult problem in genetic counseling, especially in prenatal cases. In our study, cytobands 17p11.1 to 17q11.1 appear to be potentially inert to a copy number gain.^15^

To date, few cases of prenatally diagnosed XX males have been described, as all previous cases have been detected by invasive testing.^16^ A case report involving a normal female by NIPT with male external genitalia in a routine fetal morphology assessment was first reported by Mansfield et al^17^In this work, a fetus with an unbalanced translocation involving the short arms of the X and Y chromosomes was described. Very recently, a series of cases have been reported on sex discordance following NIPT that highlights the many potential and complex explanations for these discordant results.^18^ A discrepancy in sex determination between chromosomal results and phenotypic manifestation is usually evident in the neonatal period or later at puberty. With the increasing use of NIPT for common autosomal and sex abnormalities, more gender discordance should be identified prenatally. Consequently, the frequency of sex discordant results could be equivalent to the incidence of the common trisomies and sex chromosome aneuploidies routinely screened for with NIPT, as previously reported.^18^

Since cffDNA contains maternal DNA and fetal DNA, intrinsic biological factors including maternal somatic mosaicism, undiagnosed maternal SCA, and maternal copy‐number imbalance could affect the accuracy of NIPT. These are possible limitations that could affect the correct identification of sex chromosome abnormalities via NIPT. The present case demonstrates the clinical utility of cffDNA‐based screening in the differential diagnosis of cases whose NIPT results for sex chromosome aneuploidy are discordant with ultrasound screening or fetal karyotyping. Discordance between NIPT gender results and the phenotypic features requires further prenatal and postnatal investigation of the fetus as well as maternal karyotyping to rule out maternal contributions, such as mosaicism. In our case, further prenatal cytogenetic molecular analyses allowed us to identify the presence of the *SRY* gene, which indicates normal external genitalia and masculinization^19^ and the absence of spermatogenic genes on Yq (AZFa, AZFb, and AZFc), which plays a role in azoospermia.^20^

It is essential to have a correct diagnosis of SCAs in the neonatal period, specifically to prescribe early therapy for babies with 45,X or 47,XXY syndromes. Hence, earlier screening with NIPT could improve the prognosis for many SCA cases as well as in cases of sex discordance disorder.

## CONFLICT OF INTEREST

The authors have no conflicts of interest to declare.

## AUTHOR CONTRIBUTIONS

LDF: conceived and designed the study, drafted the manuscript, and critically revised the manuscript. GS: enrolled the patients, performed and coordinated the laboratory experimental and data acquisition, and critical revised the manuscript; TS: carried out karyotype analysis and FISH. SA: performed genetic counseling, provided clinical information, and critically revised the manuscript. RR: carried out SNP array and microdeletion analyses. PS: carried out NIPT analysis and interpreted the data. AF: critically revised the article and gave final approval of the version to be published.
